# A worm gel-based 3D model to elucidate the paracrine interaction between multiple myeloma and mesenchymal stem cells

**DOI:** 10.1016/j.mtbio.2019.100040

**Published:** 2020-01-07

**Authors:** Renza Spelat, Federico Ferro, Paolo Contessotto, Nicholas J. Warren, Grazia Marsico, Steven P. Armes, Abhay Pandit

**Affiliations:** a CÚRAM, SFI Research Centre for Medical Devices, National University of Ireland Galway, Galway-H91 TK33, Ireland; b Department of Chemistry, University of Sheffield, Sheffield, South Yorkshire S3 7HF, United Kingdom

**Keywords:** Multiple myeloma, Mesenchymal stem cells, Thermoresponsive hydrogel, 3D model, Paracrine interaction

## Abstract

Multiple myeloma (MM) is a malignancy of terminally-differentiated plasma cells that develops mainly inside the bone marrow (BM) microenvironment. It is well known that autocrine and paracrine signals are responsible for the progression of this disease but the precise mechanism and contributions from single cell remain largely unknown. Mesenchymal stem cells (MSC) are an important cellular component of the BM: they support MM growth by increasing its survival and chemo-resistance, but little is known about the paracrine signaling pathways. Three-dimensional (3D) models of MM-MSC paracrine interactions are much more biologically-relevant than simple 2D models and are considered essential for detailed studies of MM pathogenesis.

Herein we present a novel 3D co-culture model designed to mimic the paracrine interaction between MSC and MM cells. MSC were embedded within a previously characterized thermoresponsive block copolymer worm gel that can induce stasis in human pluripotent stem cells (hPSC) and then co-cultured with MM cells. Transcriptional phenotyping of co-cultured cells indicated the dysregulation of genes that code for known disease-relevant factors, and also revealed IL-6 and IL-10 as upstream regulators. Importantly, we have identified a synergistic paracrine signaling pathway between IL-6 and IL-10 that plays a critical role in sustaining MM cell proliferation.

Our findings indicate that this 3D co-culture system is a useful model to investigate the paracrine interaction between MM cells and the BM microenvironment *in vitro*. This approach has revealed a new mechanism that promotes the proliferation of MM cells and suggested a new therapeutic target.

## 1 Introduction

Multiple myeloma (MM) is a B-cell neoplasia that develops within the bone marrow (BM) microenvironment that sustains the induction, selection and expansion of cancer cells [[1], [2], [3]]. The abnormal growth of malignant plasma cells is strongly dependent on autocrine and paracrine loops involving the surrounding microenvironment that supports MM cell proliferation, survival, migration and chemo-resistance [4,5].

Many new autocrine and/or paracrine myeloma growth factors have been identified [6,7]. However, the contribution of individual cell types is still largely unknown, making it difficult to understand intercellular communication signaling in MM. Mesenchymal stem cells (MSC) are a key cellular component of the BM; they are capable of self-renewal and differentiation and are known to play multiple roles in tumor progression [8,9]. MSC support MM cell growth, survival and drug resistance through paracrine circuits [10,11], but little is known about the respective mechanisms.

Comparison of MSC derived from the BM of myeloma patients (MM-MSC) with those from normal donors (ND-MSC) suggests that MM-MSC provide efficient support for MM cell migration [12]. Within hours of co-culture with MM cells, normal MSC can become *in vitro* MM-MSC that display a phenotype similar to that of patient-derived MM-MSC [13]. This phenotype is characterized by abnormally high production of soluble regulatory factors such as cytokines, chemokines and growth factors that play a fundamental role in the crosstalk between MM cells and MSC [14].

To investigate the role of this crosstalk in the pathogenesis and progression of MM, appropriate *in vitro* models are considered essential. However, current MM *in vitro* studies mainly involve two-dimensional (2D) cell culture [15], which are unable to reproduce the desired physiological response. Moreover, animal models are inadequate predictors for human MM disease and drug response owing to their inter-species differences. These problems highlight the urgent need for more biologically-relevant three-dimensional (3D) models of myeloma growth. The importance of using 3D models to elucidate physiological interactions has long been recognized in the field of tissue engineering [16,17], but this important concept has only recently been introduced to study MM pathogenesis and progression [18,19].

In view of these considerations, we have developed a novel co-culture system between BM-derived MSC and MM cells to mimic the *in vivo* paracrine interaction. For this purpose, we embedded MSC within a wholly synthetic, highly biocompatible thermoresponsive hydrogel [20]. More specifically, a poly(glycerol monomethacrylate)-block-poly-(2-hydroxypropyl methacrylate) (PGMA−PHPMA) diblock copolymer is synthesized via RAFT (reversible addition-fragmentation chain transfer) aqueous dispersion polymerization in the form of worm-like micelles, which interact to afford a soft, free-standing gel via multiple inter-worm contacts [21]. Importantly, this well-characterized formulation [22,23] is a hydroxyl-rich mucin-mimicking hydrogel capable of maintaining pluripotent stem cells in their dormant, non-proliferative G0 state [24]. Such quiescence is reversible and is an intrinsic *in vivo* property of pluripotent (and multipotent) stem cells that can be also induced *in vitro* [[25], [26], [27]].

RNA-seq transcriptomic profiling of MSC and MM cells indicated broad changes in both cell types as a consequence of co-culture, which enabled us to verify our hypothesis of a paracrine loop involving IL-6 and IL-10 that sustains MM cell proliferation.

Overall, this study provides new insights for understanding the effect of paracrine signals between MSC and myeloma cells, and highlights the pivotal role played by MSC in the pathophysiology of MM.

## 2 Material and methods

### 2.1 Cell culture

MM cell lines RPMI-8226, MM1S and JJN3 (kindly provided by Prof. Michael O'Dwyer, National University of Ireland, Galway, IE) were cultured in RPMI 1640 medium (Sigma-Aldrich, St Louis, MO, http://www.sigmaaldrich.com) supplemented with 10% FBS (Fetal bovine serum, Gibco, Thermo Scientific, Waltham, MA, http://www.thermofisher.com), 60 mg/ml penicillin, and 100 mg/mL streptomycin (Sigma-Aldrich) and incubated at 37 °C in a humidified atmosphere containing 5% CO_2_.

MSC derived from human bone marrow aspirates (kindly provided by Prof. Dimitrios Zeugolis, National University of Ireland, Galway, IE) were isolated using a protocol previously described [28], and cultured in complete medium (MEM alpha, GlutaMAX supplemented with 10% fetal bovine serum and 1% penicillin/streptomycin), and maintained at 37 °C in a humidified atmosphere containing 5% CO_2_.

### 2.2 Protocol for the synthesis of PGMA_52_ Macro-CTA

PGMA_52_ (G_52_) was synthesized as follows: CPDB RAFT agent (0.864 g, 3.9 mmol) and GMA monomer (25.0 g, 156.1 mmol) were weighed into a 100 mL round-bottomed flask and purged under N_2_ for 30 min. ACVA initiator (218.6 mg, 0.78 mmol; CTA/ACVA molar ratio = 5.0) and anhydrous ethanol (49.6 mL; previously purged with N_2_ for 30 min) were then added, and the resulting red solution was degassed for a further 10 min. The flask was subsequently sealed and immersed into an oil bath set at 70 °C. After 100 min, the polymerization was quenched by opening to air, immersing in liquid nitrogen for 30 seconds followed by dilution with methanol (100 mL). A final GMA conversion of 78% was determined by ^1^H NMR analysis. The methanolic solution was precipitated into a ten-fold excess of dichloromethane. After filtering and washing with dichloromethane, the crude polymer was dissolved in water and the residual dichloromethane was evaporated under vacuum. The resulting aqueous solution was freeze-dried overnight to yield a pink powder. ^1^H NMR analysis indicated a mean degree of polymerization of 52 for this PGMA macro-CTA. DMF GPC indicated a number average molecular weight, *M*_n_ of 14 800 and a molar mass dispersity, *M*_w_/*M*_n_ of 1.10 (Fig. S1, Supporting Information).

### 2.3 Protocol for the synthesis of PGMA_52_ - PHPMA_122_ diblock copolymer

PGMA macro-CTA (0.726 mmol, 6.74 g), hydroxylpropyl methacrylate (92.0 mmol, 13.26 g Target DP = 130) and ACVA (0.143 mmol, 0.040 g, 5:1 CTA:initiator) were weighed into a 1000 mL round bottomed flask fitted with a two-necked flange lid and an overhead stirrer. 180 mL deionized water was added to the flask and the stirrer was turned on so as to maintain the solid components in suspension while dissolving. During this process, a nitrogen needle attached to a schlenk line was inserted through a rubber septum placed in one of the reactor ports and nitrogen was bubbled through the reaction mixture for 45 min in order to remove traces of oxygen. After this period, the nitrogen needle was raised above the surface of the liquid and the flask was immersed into an oil bath set to 70 °C. After three hours the reaction was terminated by opening to air and immersing the flask into an ice-bath. Once cooled, the viscous liquid was transferred into dialysis tubing (3000 MWCO) and dialysed for one week, with twice daily water changes to ensure all small-molecule impurities were removed. The dispersion was then transferred into a 1000 mL round bottomed flask and placed in the freezer overnight before freeze drying to form a pink powder. ^1^H NMR analysis indicated a copolymer composition of PGMA_52_-PHPMA_122_ based on a HPMA monomer conversion of 94% (Fig. S2, Supporting Information). DMF GPC indicated a number average molecular weight, *M*_n_ of 35 500 and a molar mass dispersity, *M*_w_/*M*_n_ of 1.14 (Fig. S1, Supporting Information).

### 2.4 ^1^H NMR spectroscopy

^1^H NMR spectra were recorded in CD_3_OD using a 400 MHz Bruker Avance-400 spectrometer (64 scans averaged per spectrum).

### 2.5 Gel permeation chromatography (GPC)

Copolymer molecular weights and molar mass dispersities were determined using an Agilent 1260 Infinity DMF GPC operating at 60 °C and equipped with two Polymer Laboratories PL gel 5 μm Mixed-C columns and a guard column. The GPC eluent was HPLC grade DMF containing 10 mM LiBr at a flow rate of 1.0 mL min^−1^. DMSO was used as a flow-rate marker. The refractive index detector was used for calculation of molecular weights and molar mass dispersities by calibration using a series of ten near-monodisperse poly(methyl methacrylate) standards (M_n_ = 625− 618 000 g mol^−1^).

### 2.6 Oscillatory rheology studies

An AR-G2 rheometer equipped with a variable temperature Peltier plate and a 40 mm 2° aluminum cone was used in oscillatory mode to measure loss modulus (*G*″) and storage modulus (*G*′) as a function of temperature. Temperature sweeps were conducted at an applied strain amplitude of 1.0% and at an angular frequency of 1 rad s^−1^. Measurements were recorded at 1 °C intervals, allowing 3 min for thermal equilibration between points. The critical gelation temperature (CGT) was noted as the temperature at the crossover point for *G’* and *G”* (Fig. S3, Supporting Information)*.*

### 2.7 Three-dimensional cell culture

A 10% w/v PGMA_52_-PHPMA_122_ worm gel dispersed in the MSC culture medium was cooled to 4 °C to induce degelation via a worm-to-sphere transition, affording a free-flowing dispersion of copolymer spheres. This cold, low-viscosity fluid was then ultrafiltered using a sterile 0.20 μm syringe filter. Syringes and filters were stored at −20 °C for at least 1 h prior to ultrafiltration to prevent gelation on contact. The resulting sterilized copolymer worm gel was then used to encapsulate MSC at a concentration of 10^6^ cells/ml.

### 2.8 Cell viability assay

The alamarBlue® (Invitrogen, Carlsbad, NY, http://www.invitrogen.com) assay was used to evaluate the metabolic activity of MSC in the 3D cell culture system. At each time point, 10% alamarBlue® in Phosphate Buffer Saline (PBS) was added to assess the cell metabolic activity after 2, 4 and 7 days, the absorbance was measured at the wavelengths of 550 nm and 595 nm using a microplate reader (Varioskan Flash, ThermoScientific). The level of metabolic activity was determined using the simplified method of calculating % of reduction, according to the supplier's protocol. The LIVE/DEAD® assay (Molecular Probes, Life Technologies, Eugene, OR) was also used to visualize the distribution of living and dead cells in the gel at the same time points. Cell viability was evaluated using calcein AM and ethidium homodimer-1 (EthD-1). The calcein binds to live cells and produces green fluorescence, while EthD-1 binds to dead cells and produces red fluorescence. The staining solution was prepared in PBS at a concentration of 2 μM calcein and 4 μM EthD-1. Fluorescence images were recorded using a Fluoview 1000 Confocal Microscope (Olympus).

### 2.9 Cell proliferation assays

Cell proliferation was determined using a PicoGreen® fluorescent DNA quantification kit (Molecular Probes, Life Technologies) for both 3D and 2D cultures at 2, 4 and 7 days as described by the manufacturer. Recovery of cells from 3D cultures was obtained by centrifuging the gel in PBS at 4 °C. Briefly, 100 μl DNA samples were incubated with 100 μl diluted (1:200) PicoGreen® reagent in Tris EDTA (TE) buffer in a 96-well opaque, flat-bottomed assay plate. Fluorescence was read at 485 nm and 525 nm, and then compared with a DNA standard curve. For inhibition experiments, MM cell cultures were treated with combinations of IL-6, IL-10 and blocking antibodies (R&D Systems, Minneapolis, MN) in serum-free media at a concentration of 100 ng/ml for 24 h.

Proliferation of cells after treatment with IL-6 and IL-10 was tested using PicoGreen® and BrdU assay (Sigma-Aldrich), which detects incorporation of 5-bromo-2′-deoxyuridine in proliferating cells. Plates were read at 450 nm wavelength.

### 2.10 RNA isolation

RNA was isolated from MSC and MM cells both mono-cultured and co-cultured for 12 h using an Arcturus® PicoPure® RNA isolation kit (Applied Biosystems, Waltham, MA, http://www.thermofisher.com) according to the manufacturer's instructions. RNA quality and quantity were measured using 2100 Bioanalyser and RNA 6000 Pico Kit, as per standard protocol (Agilent Technologies, Santa Clara, CA). Samples with a RIN (RNA integrity number) value > 9 were used for RNA-seq analysis.

### 2.11 RNA-sequencing

After cell retrieval and total RNA extraction as described above, samples were submitted to Genewiz (South Plainfield, NJ, https://www.genewiz.com), where mRNA enrichment and library preparation were performed. Individual samples were bar-coded and run on a HiSeq 2500 sequencer (Illumina, San Diego, CA). The data presented here represents the sequencing of three pooled replicates from three independent experiments per sample.

### 2.12 Ingenuity Pathway Analysis

RNA-seq data were analyzed using Qiagen's Ingenuity® Pathway Analysis software (IPA®, Qiagen Redwood City, CA, www.qiagen.com/ingenuity). Two parameters were taken into consideration to determine the significance of the association between the input data set and the functions or pathways: (1) a ratio of the number of genes from the data set that map to the function/pathway divided by the total number of genes that map to the function/pathway and (2) a *p-*value calculated using Fischer's exact test determining the probability that the association between the genes in the dataset and the function/pathway is explained by chance alone. DAVID Gene Functional Classification Tool (http://david.abcc.ncifcrf.gov) was used for the functional enrichment analyses on IL-6 and IL-10 target genes.

### 2.13 Cytokine protein array and ELISA

Supernatants from mono/co-cultures were harvested after 24 and 48 h and analyzed with a human inflammation cytokine antibody array (RayBio® Inflammation Antibody Array C series; Raybiotech Parkway, GA) that enables the simultaneous detection of 40 cytokines. All analyses were performed according to the manufacturer's guidelines. Concentrations of IL-10 and IL-6 in mono/co-culture supernatants and lysates were measured using ELISA kits (R&D Systems), as per standard protocol. Colorimetric absorbance was recorded at 450 nm.

### 2.14 PathScan® sandwich immunoassay

This commercially-available immunoassay (Cell Signaling Technology, Danvers, MA) was used according to the manufacturer's instructions. Briefly, cell lysates were harvested after 24 h of mono and co-culture using the buffer provided with the kit. The Array Blocking Buffer was added to each well and incubated for 15 min at room temperature. Subsequently, the lysate was added to each well and incubated overnight at 4 °C. After washing, the detection antibody cocktail was added to each well and incubated for 1 h at room temperature. Horseradish peroxidase (HRP)-linked streptavidin was added to each well and incubated for 30 min at RT. The slide was then covered with Clarity™ ECL (Bio-Rad) and images were captured using a digital imaging system (Bio-Rad). Image J version 1.46r (NIH, http://imagej.nih.gov/) was used for spot quantifications.

### 2.15 Statistical analysis

All data are expressed as mean ± standard deviation of triplicate samples. Comparisons between multiple groups were performed using one-way ANOVA and Tukey's post hoc test. All analyses were performed with GraphPad Prism 5 (San Diego, CA). Differences between two datasets were considered significant if P < 0.05.

## 3 Results

### 3.1 3D model characterization

#### 3.1.1 Establishment of MM-MSC co-culture system using diblock copolymer worm gel

**Fig. 1:**
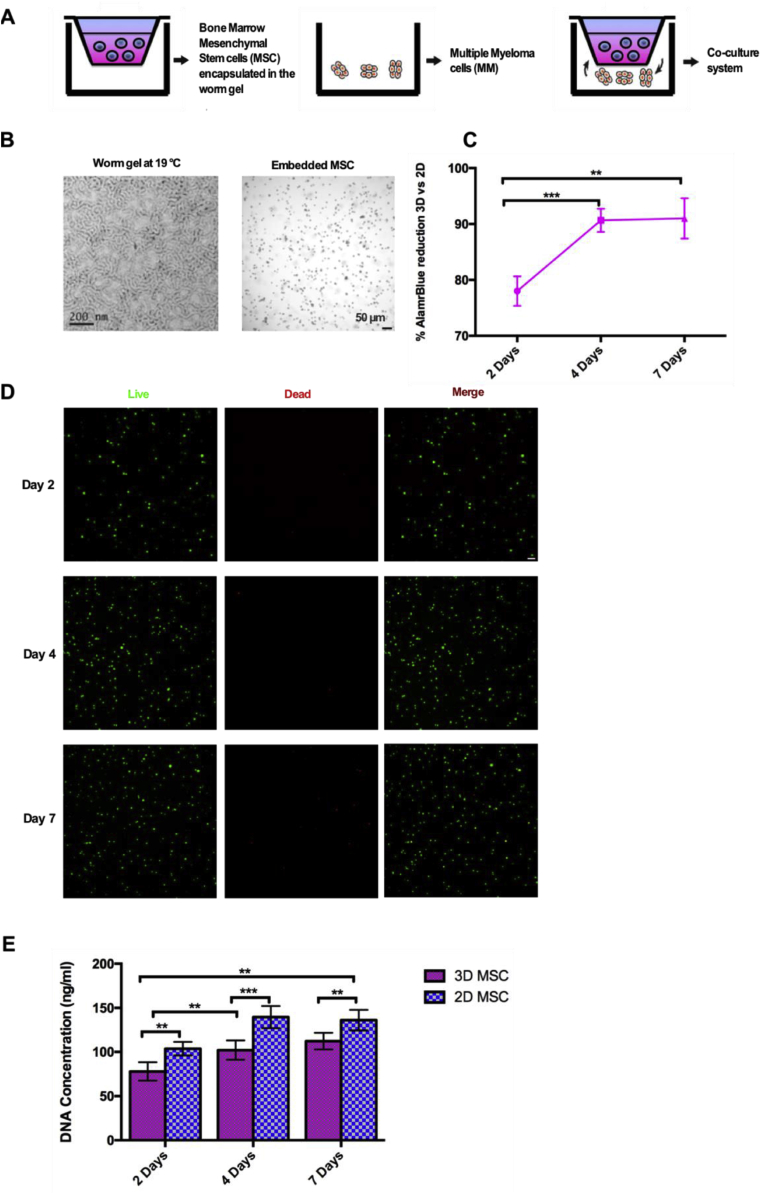
**Co-culture system and MSC encapsulation characterization**. A) Schematic representation of the co-culture system. B) Electron microscopy image of the worm gel (left) and contrast phase image of encapsulated MSC (right). C) Cellular metabolic activity for encapsulated MSC expressed as 3D vs 2D percentage difference. D) Representative confocal Z-stack images of encapsulated MSC stained with calcein AM (live, green) and ethidium homodimer (dead, red) viability staining kit at 2, 4 and 7 days of culture. Scale bar = 50 μm. E) Cellular proliferation determined by PicoGreen® assay after recovering the DNA from cells in both 3D and 2D culture. (N = 3 independent experiments, *p < 0.05, * *p < 0.01, * * *p < 0.001).

A thermoresponsive PGMA_52_-PHPMA_122_ worm gel with a critical gelation temperature of 19 °C, as judged by oscillatory rheology studies (Fig. S3, Supporting Information) [20], was used to embed MSC in a co-culture system with RPMI 8226 Multiple Myeloma cell line (MM) to study the paracrine interaction. This 3D model is illustrated in Fig. 1A. Briefly, MSC were encapsulated within a 10% w/v thermoresponsive aqueous copolymer dispersion and, before gelation, added to a transwell and transferred to a 24-well plate containing MM cells. The following systems were examined: (i) MSC in 3D culture alone, (ii) MSC in 3D co-cultured with MM cells and (iii) MM cells alone.

#### 3.1.2 MSC morphology, viability and proliferation within PGMA_52_ - PHPMA_122_ worm gel

After MSC encapsulation within the worm gel, the cell morphology was evaluated using a confocal microscope. A rounded morphology was observed and the MSC were uniformly distributed throughout the gel (Fig. 1B). Cellular metabolic activity was examined using the alamarBlue® assay. These experiments indicated a significant increase between day 2 (78.0 ± 2.6%) and day 4 (90.7 ± 2.1%) but remained stable at day 7 (91.0 ± 3.6%) (Fig. 1C). Unseeded worm gels alone were used as negative controls and tissue culture plastic (2D) seeded MSC were used as a positive control. The LIVE/DEAD® assay was employed to visualize the distribution of living and dead cells after 2, 4 and 7 days in the 3D cell culture system. A few dead cells were observed after 7 days but the vast majority of cells remained alive at this time point (Fig. 1D). To assess the rate of proliferation, the cell-encapsulated worm gels were cooled to 4 °C to induce degelation and then centrifuged to isolate the cells for further analysis. DNA content was quantified using the PicoGreen® assay after 2, 4 and 7 days and compared to MSC grown in a 2D cell culture model (Fig. 1E). While the DNA concentration from encapsulated cells increased across all time points (78.0 ± 10.4 ng/ml at day 2, 102.1 ± 10.9 ng/ml at day 4 and 112.3 ± 9.4 ng/ml at day 7), the rate of proliferation from 3D-seeded cells was significantly lower than that of 2D-seeded cells (103.7 ± 7.7 ng/ml at day 2, 139.6 ± 12.6 ng/ml at day 4 and 136.0 ± 11.7 ng/ml at day 7). This is consistent with the metabolic activity data. We have previously demonstrated that this polymer exhibits comparable gel moduli to natural mucins, and that this mucin-mimicking 3D gel is able to maintain Pluripotent Stem Cells (PSC) in a G_0_ quiescent state, without inducing proliferation or differentiation commitment, but maintaining the potential to do it. These properties make our system unique when compared to previous ones, because of the state in which MSC are maintained, owing to the relationship between the cell-material interaction and cell fate determination [24].

### 3.2 Differential gene expression profiling and pathway analysis of MSC and MM cells in co-culture

**Fig. 2:**
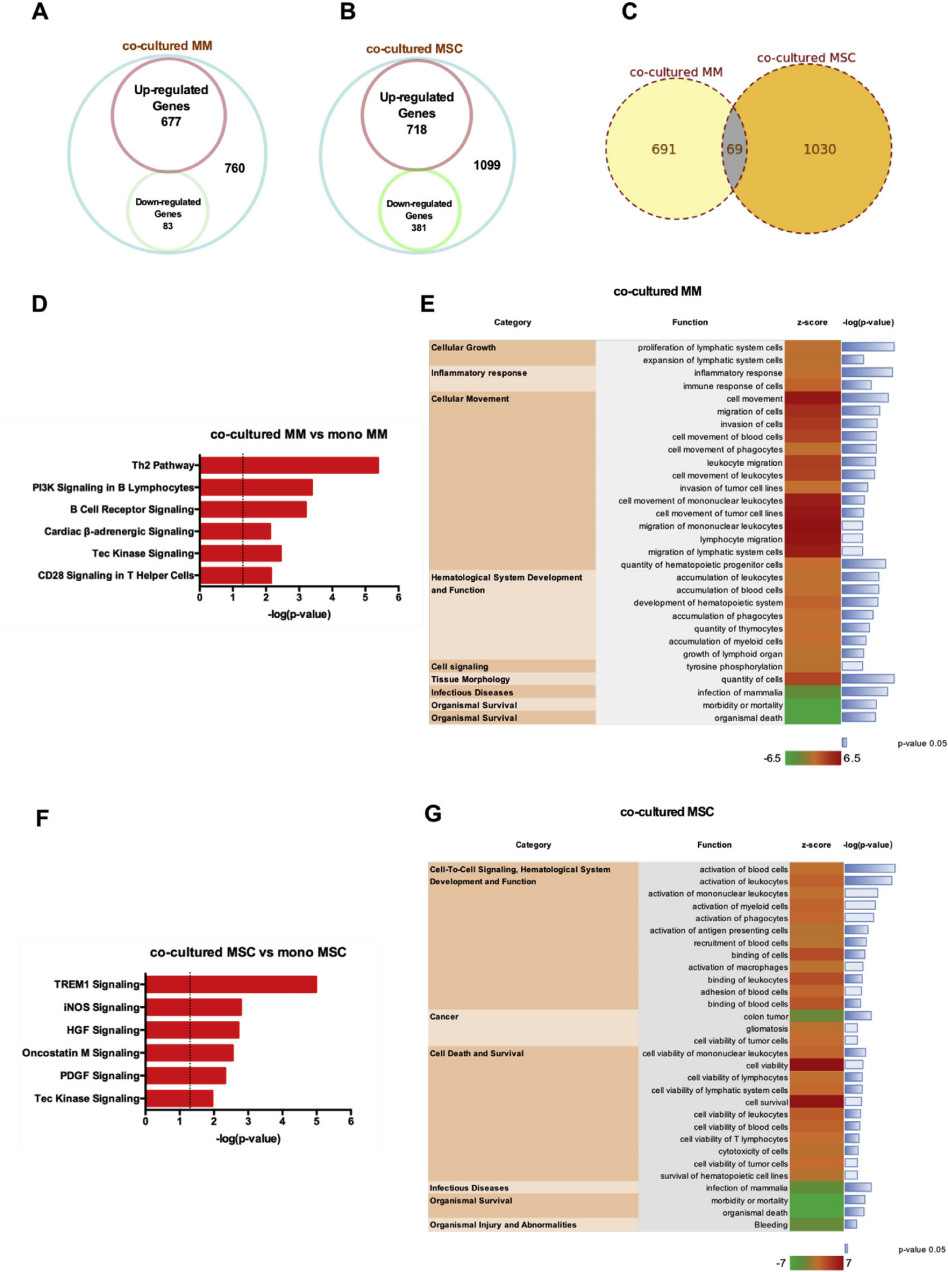
**RNA-seq analysis of co-cultured MSC and MM cells**. A) Venn Diagram of differentially expressed (DE) genes in co-cultured MM cells, B) MSC and C) common DE genes. Genes with a fold change greater than two (the absolute value of log_2_ fold change greater than unity) and an adjusted *p*-value less than 0.05 were considered to be DE genes. D) The most activated and statistically significant canonical pathways in co-cultured MM vs. mono-cultured MM cells and F) co-cultured MSC vs. mono-cultured MSC identified using the IPA® software are listed according to their *p-*value (-log); the threshold -log(*p*-value) = 1.3 corresponds to a *p*-value of 0.05. E) Functional enrichment analysis and predicted activation/inhibition statuses in co-cultured MM vs. mono-cultured MM cells and G) co-cultured MSC vs. mono-cultured MSC identified using the IPA® software; threshold: activation z-score = 2, inhibition z-score = −2; Fisher's Exact Test. (N = 3 pooled replicates from three independent experiments).

To investigate the molecular consequences of co-culture, RNA-seq analysis was performed on both mono- and co-cultured MM and MSC. We applied a 5% false discovery rate (FDR), an adjusted *p*-value ≤ 0.05, and fold change ≥ 1.0 log2 for up- or down-regulation as the criteria to define differentially-expressed (DE) genes. As per the above criteria, 760 genes were DE in MM cells following co-culture with MSC. Of these, 667 genes were up-regulated, whereas 83 genes were down-regulated (Fig. 2A); in MSC co-cultured with MM, 1099 genes were DE, and, of these, 718 were up-regulated and 381 were down-regulated (Fig. 2B); common DE genes were 69 (Fig. 2C). Among the most differentially-expressed genes in co-cultured MM cells, we identified a chemokine (C-X-C motif) receptor to be highly over-expressed, with a fold change (FC) of 182.6, namely CX3CR1. The CX3CL1/CX3CR1 axis has been previously implicated in the interaction between multiple myeloma cells and the bone microenvironment, and also associated with cell survival and disease progression [29]. Interestingly, we also found the transcription factor IRF4 (interferon regulatory factor 4) to be up-regulated in the co-culture condition (FC = 19.54); this transcription factor has been reported to be essential for the survival of MM cells [30]. Moreover, we found several genes involved in the positive regulation of B cell activation and proliferation such as Bcl-2 (B-cell lymphoma 2, FC = 2.38), CD38 (FC = 10.38), NCKAP1L (Nck-associated protein 1-like, FC = 8.94) and TLR9 (Toll-like receptor 9, FC = 29) to be over-expressed in co-culture. On the other hand, MSC in co-culture with MM cells showed up-regulation of several chemokine ligands (CXCL6, FC = 3.66; CXCL8, FC = 8.17; CXCL9, FC = 3.09; CXCL10, FC = 2.85; CCL2, FC = 4.56 and CCL7, FC = 15,62) reported to regulate cell trafficking and migration of immune cells [[31], [32], [33]], and interleukins (IL-7, FC = 2.43; IL-10, FC = 13.83; IL-22, FC = 3.37; IL-32, FC = 3.06) with immuno-modulatory properties [[34], [35], [36], [37]].

We next performed pathway and functional enrichment analyses using IPA® to identify pathways that were altered by the co-culture condition. The most activated pathways in MM co-cultured with MSC were mainly related to immune response and B cell activation (Fig. 2D), e.g. PI3K signaling in B lymphocytes and B cell receptor signaling. In contrast, the Triggering Receptor in Myeloid cell 1 (TREM1) signaling, a proinflammatory immune response and a regulator of Toll-Like Receptors (TLR) signaling, was the most significant activated pathway for MSC (Fig. 2F).

In addition, we conducted a functional enrichment analysis to identify the most statistically-significant functional categories associated with DE genes in co-culture. We found that the functional categories that were more enriched in MM cells were relative to “inflammatory response”, “cellular growth” and “cellular movement” (Fig. 2E), whereas for MSC the functional categories were “cell-to-cell signaling”, “hematological system development and function”, “cancer” and “cell death and survival” (Fig. 2G).

### 3.3 Upstream regulator analysis reveals activated IL-6 and IL-10 signaling in co-cultured MM cells

**Fig. 3:**
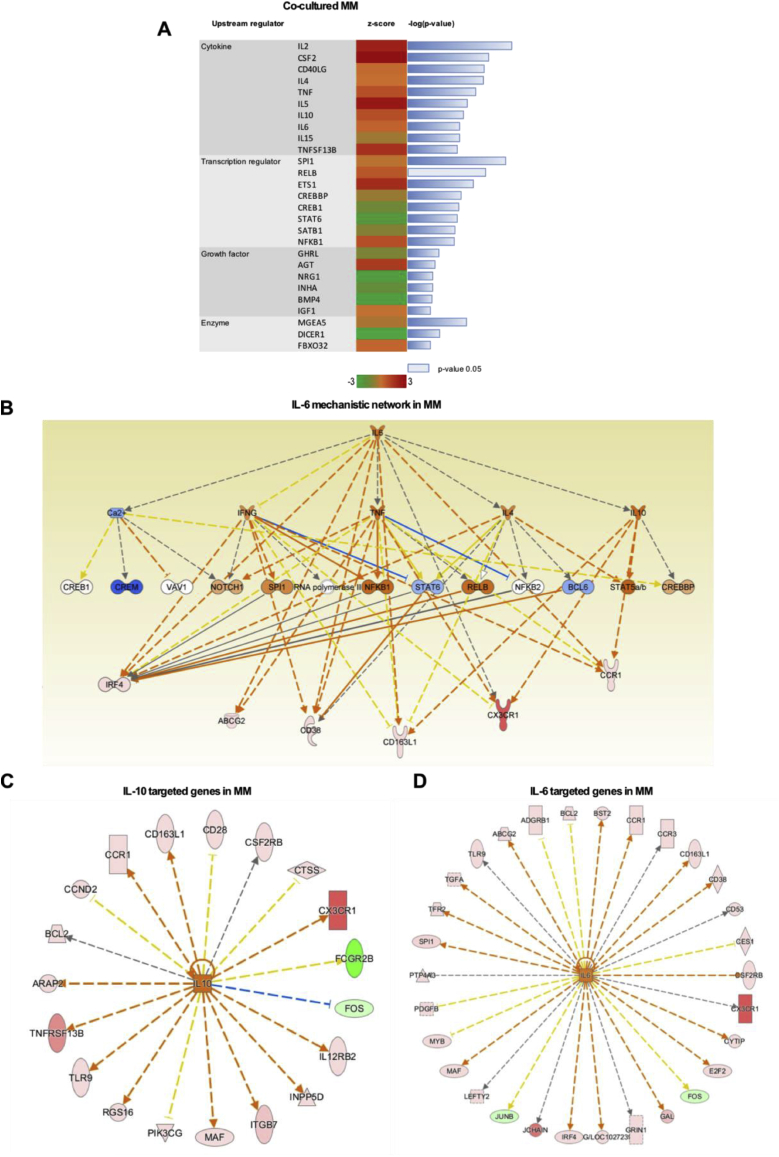
Upstream regulator analysis of the differentially expressed genes in co-cultured MM cells. A) Upstream regulators in the co-cultured MM cells identified using the IPA® Upstream Regulator Analysis (URA) tool. B) IL-6 mechanistic network from IPA® showing network regulators and some of the differentially expressed downstream genes (IRF4, ABCG2, CD38, CD163L1, CX3CR1, CCR1). Shades of red indicate the degree of upregulation, while shades of green represent the degree of downregulation. The edges connecting the nodes are colored orange when leading to activation of the downstream node, blue when leading to inhibition, yellow if the findings underlying the relationship are inconsistent with the state of the downstream node, and grey if the effect is not predicted. Pointed arrowheads indicate that the downstream node is expected to be activated, while blunt arrowheads indicate that the downstream node is expected to be inhibited. IL-10C) and IL-6 D) downstream genes. (N = 3 pooled replicates from three independent experiments).

To further investigate the mechanism behind the observed changes in expression, we used the Upstream Regulator Analysis (URA) which is a tool implemented within the IPA® software to identify the molecules upstream of the genes in the dataset and their associated networks [38]. We performed URA on co-cultured MM cells to identify potential upstream regulators induced by the paracrine interaction with MSC and able to modulate different functions in MM. Using this approach for the most significant upstream regulators (Bonferroni corrected *p*-value < 0.05), we found some of the cytokines reported to be implicated with MM progression (Fig. 3A) including IL-6 and IL-10, which were reported to be involved in MM cell proliferation [39,40]. Interestingly, the causal effect of the upstream regulator IL-6 on the data set, as evidenced in the mechanistic network (Fig. 3B), was predicted to be relayed also through IL-10, which was found to be upregulated in co-cultured MSC (FC = 13.8). Moreover, this network also influences the expression of some of the overexpressed genes in MM discussed in the previous paragraph, i.e. CX3CR1, IRF4 and CCR1. In particular, MM cells display 19 DE genes that are targeted by IL-10 (Figure 3C) and 30 targeted by IL-6 (Fig. 3D). The only relationship involving inhibition that is predicted to be consistent is downregulation of the FOS proto-oncogene among the IL-10 targeted genes, in agreement with previous findings [41]. We performed functional classification analyses of these DE genes using DAVID software package [42]. This analysis indicated that the biological category “Pathways in cancer” is the most enriched for IL-10 targeted genes (Table 1) and “Cytokine-Cytokine receptor interaction” for the IL-6 targeted genes (Table 2). Several genes of these categories were up-regulated in MM cells as a result of co-culture with MSC, indicating a possible activation state. In the following sections, we focus on the validation and quantification of the hypothesized alterations suggested by the pathway and functional classification analyses.

**Table 1:** Functional annotation analysis using DAVID software of the IL10-downstream genes. Enrichment scores ≥1 are considered significant.

Biological category (KEGG_PATHWAY)	Enrichment score	Genes
Pathway in cancer	2.65	BCL2, E2F, FOS, SPI1, PDGFB, TGFA
HTLV-I infection	2	E2F, FOS, MYB, SPI1, PDGFB
Cytokine-cytokine receptor interaction	1.62	CCR1, CCR3, CX3CR1, PDGFB

**Table 2:** Functional annotation analysis using DAVID software of the IL6-downstream genes. Enrichment scores ≥1 are considered significant.

Biological category (KEGG_PATHWAY)	Enrichment score	Genes
Cytokine-cytokine receptor interaction	2.48	CCR1, CX3CR1, TNFRSF13B, CSF2RB, IL12RB2
T cell receptor signaling pathway	2.36	CCR1, FOS, PIK3CG, TLR9
B cell receptor signaling pathway	1.68	FCGR2B, FOS, INPP5D, PIK3CG

### 3.4 Crosstalk between MSC and MM cells induces IL-6 and IL-10 secretion

**Fig. 4:**
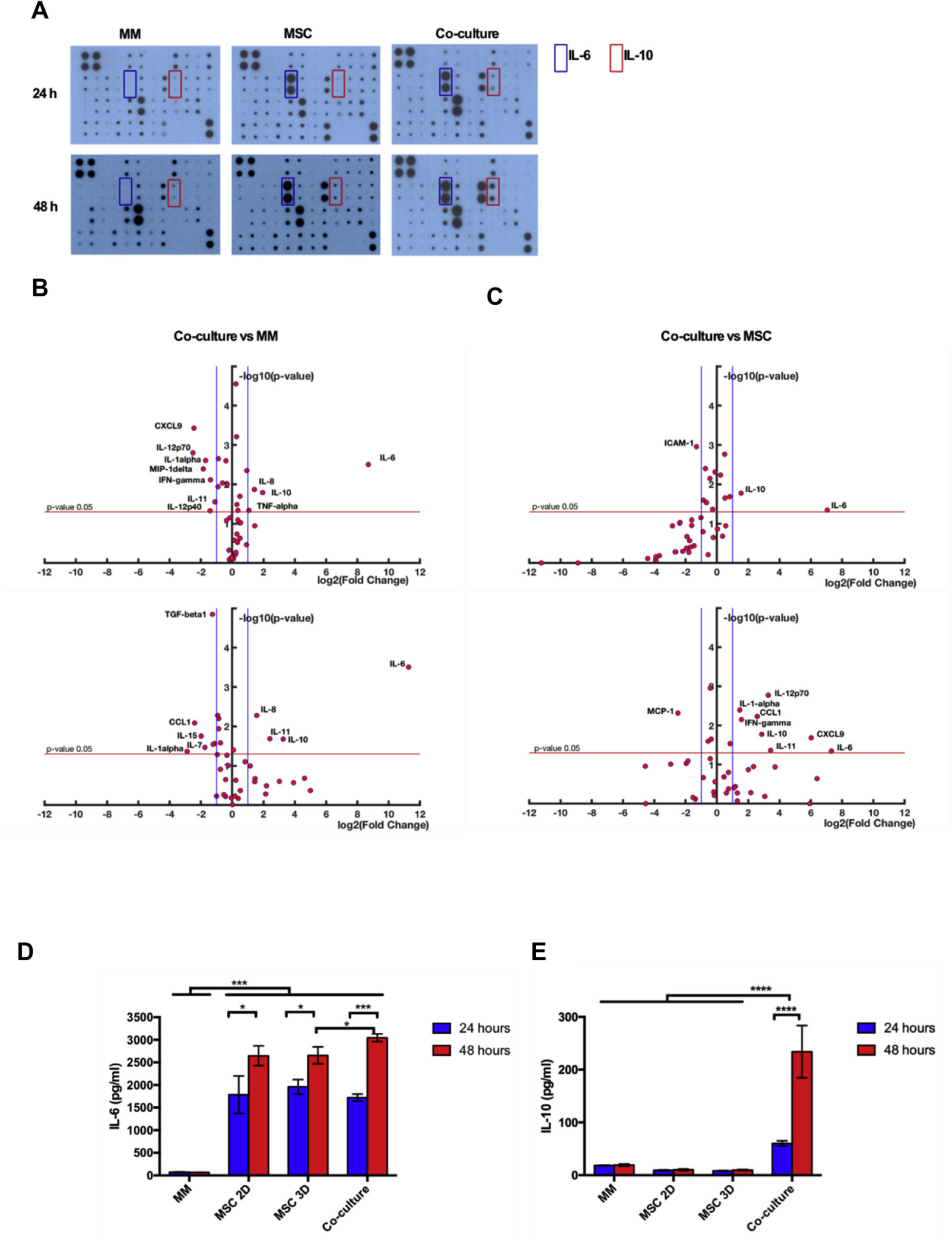
**Co-culture enhances IL-6 and IL-10 secretion**. A) Representative images of cytokine array scans performed on the culture media of MM, MSC and co-cultured cells after 24 and 48 h of culture. IL-6 and IL-10 are framed within blue and red rectangles, respectively. B) Dot plots of cytokine array quantifications of co-cultured vs mono-cultured MM cells and C) co-cultured vs mono-cultured MSC at 24 and 48 h. D) IL-6 and E) IL-10 ELISA quantification of the respective supernatants derived from MM cells, MSC in monolayer culture (MSC 2D), encapsulated MSC (MSC 3D) and MM cells plus MSC 3D (co-culture) after 24 and 48 h of culture. (N = 3 independent experiments, *p < 0.05, * *p < 0.01, * * *p < 0.001).

To better understand the crosstalk between MM and MSC cells and to elucidate how the co-culture influences the cytokine secretion profile, a cytokine protein array was conducted on media from mono- and co-culture conditions after 24 and 48 h, respectively (Fig. 4A and S4A, Supporting information). Thus, we identified a characteristic “cytokine signature” associated with the co-culture condition. In particular, we observed a significant increase in IL-6, IL-8 and IL-10 in co-culture media relative to monocultured MM after 24 and 48 h, while IL-11 only increased after 48 h (Fig. 4B). With respect to mono-cultured MSC, the co-cultured media exhibited an increase in IL-10 and IL-6 after 24 and 48 h, and CCL1, CXCL9, IL-11, IFN-γ and IL-1α only after 48 h (Fig. 4C).

The consistent upregulation of IL-6 and IL-10 suggested potential roles for these interleukins in the crosstalk between MM and MSC cells. To verify this hypothesis, we first validated the greater secretion of IL-6 and IL-10 using ELISA. As expected, a significant increase in the production of IL-6 (MM cells: 67.5 ± 1.3 pg/ml; MSC 3D: 2656 ± 188 pg/ml; co-culture: 3046 ± 84 pg/ml at 48 h) and IL-10 (MM cells: 19.3 ± 1.5 pg/ml; MSC 3D: 9.7 ± 0.7 pg/ml; co-culture: 234 ± 35 pg/ml at 48 h) was observed in co-culture (Fig. 4D and E). To understand whether MSC and/or MM cells were responsible for this induced IL-6 and IL-10 secretion, we conducted further ELISA studies on cell lysates. These additional experiments confirmed that the MSC were mainly responsible for the enhanced secretion of IL-6 and IL-10 (Figs. S5A and S5B, Supporting Information). Moreover, we have evaluated the secretion of IL-6 and IL-10 after co-culture with MSC in an additional MM cell line, MM1S. The results obtained evidenced an induction of the secretion of both cytokines after co-culture, supporting our previous findings (Figs. S5C and S5D, Supporting Information).

### 3.5 IL-6 and IL-10 synergistically sustain the proliferation of MM cells

**Fig. 5:**
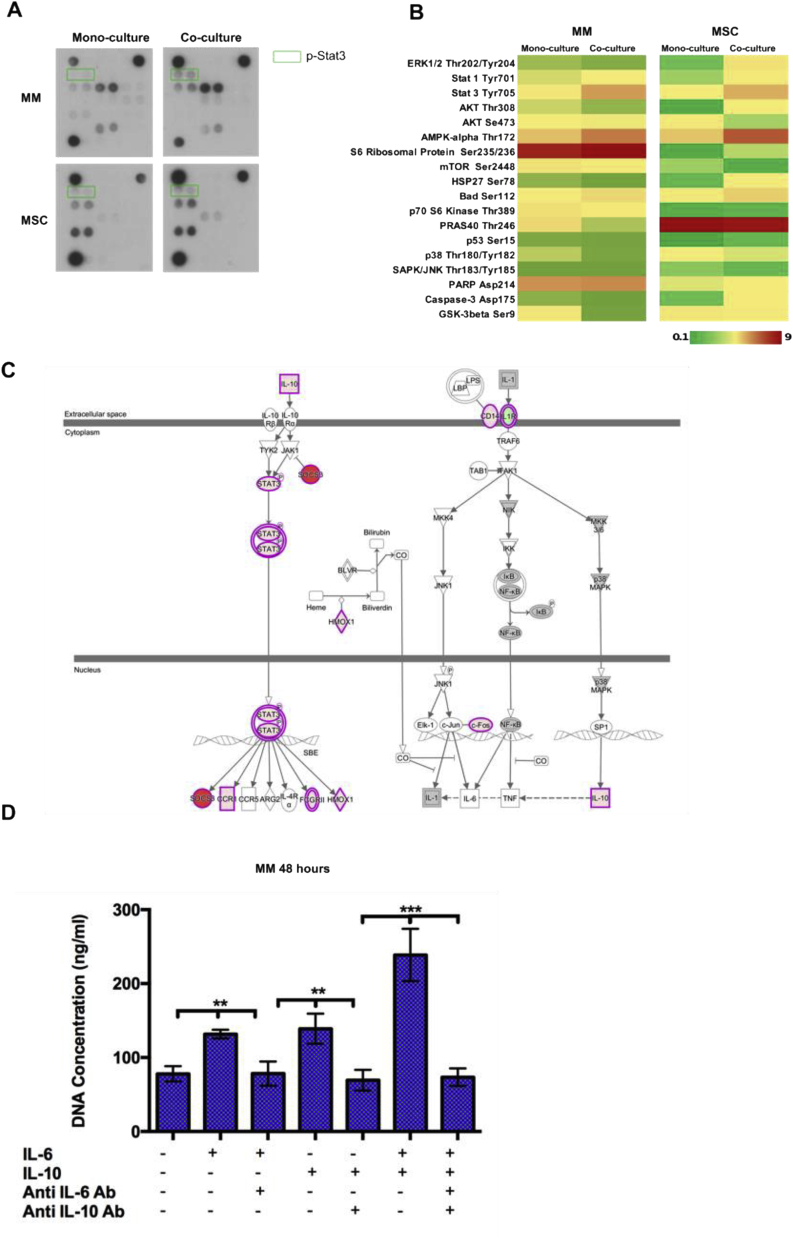
Activation of IL-6 and IL-10 signaling sustains proliferation of MM cells. A) Representative scans of PathScan® Intracellular Signaling Arrays showing Stat3 pathway activation in MSC and MM cells. B) Heatmaps of arrays quantification. C) IL-10 signaling induction in MSC indicated using IPA® software. Shades of red indicate the degree of upregulation, while shades of green represent the degree of downregulation. D) PicoGreen® proliferation assay of MM cells treated with IL-6, IL-10, neutralizing antibodies or a combination of them. (N = 3 independent experiments, *p < 0.05, * *p < 0.01, * * *p < 0.001).

Cytokines act through signal-transduction pathway activation [43]. In particular, IL-6 and IL-10 signal mainly via activation of the transcription factor Stat3 [44,45]. To assess the activation states for different pathways, cell lysates deriving from mono and co-cultures were analyzed using the PathScan® Intracellular Signaling Array Kit for the simultaneous detection of 18 important and well-characterized phosphorylated signaling molecules (Fig. 5A and S4B, Supporting Information). These data confirmed increased phosphorylation of Stat3 in both MSC and MM cells after co-culture (Fig. 5B). Moreover, IL-10 signaling induction through Stat3 activation was also supported by IPA® analysis (Fig. 5C).

In cancer cells, aberrant IL-6 signaling and constitutive activation of Stat3 are closely related to cell proliferation and drug resistance [46]. To investigate whether IL-6 and IL-10 secreted by MSC support MM cells proliferation by paracrine stimulation, we first compared MM cells proliferation in mono and co-culture to examine whether there was any evidence for enhanced proliferation under the latter conditions. We observed an increase in the rate of proliferation in MM cells (Fig. S6A, Supporting Information), but not in MSC (Fig. S6B, Supporting Information) as a consequence of co-culture. Moreover, IL-6 and IL-10-neutralizing antibody treatments reduced the proliferation of co-cultured MM cells (Fig. S6C, Supporting Information). Consistently, exogenous IL-6 (100 ng/ml) and IL-10 (100 ng/ml) significantly increased the proliferation of mono-cultured MM cells, as demonstrated by PicoGreen® and BrdU assays (Figs. S6D–E, Supporting Information).

Given that IL-6 has been reported as a proliferative factor for MM cells [47], is overexpressed by MSC after co-culture, and also signals through IL-10, we explored the possibility that IL-6 and IL-10 have a synergistic effect on the proliferation of MM cells. Cell proliferation assays confirmed a significant additional increase after concomitant treatment with IL-6 and IL-10, suggesting that IL-10 works in tandem with IL-6 to sustain MM cell proliferation, while neutralizing antibodies markedly reduced the rate of proliferation of MM cells (Fig. 5D).

To further validate these results, we analyzed the proliferation rate after treatment with IL-6 and IL-10 in an additional MM cell line, JJN3 cells, obtaining similar results to RPMI-8226 cells, and suggesting that IL-6 and IL-10 can be considered as key players in MM (Figs. S6F–G, Supporting Information).

## 4 Discussion

Investigation of the paracrine mechanisms involved in MM pathogenesis, progression and resistance to treatments requires the development of suitable *in vitro* models to determine the individual contributions of specific cell types to the overall response [19]. In this study, we focused our attention on the contribution of MSC, an essential element of the BM niche that profoundly influences the behavior of MM cells [10,48]. Specifically, we have developed a co-culture system that mimics the paracrine interaction between MSC and MM cells, while using a 3D (rather than 2D) culture system to more accurately represent multiple myeloma growth. Previously, we reported the characterization of a wholly synthetic thermoresponsive diblock copolymer hydrogel that offers a decisive advantage over commercial protein-based gels [20] because it can maintain hPSC in their quiescent state [24], which is similar to the situation encountered *in vivo*. Accordingly, encapsulation of MSC within this thermoresponsive copolymer hydrogel enabled us to evaluate a co-culture system that provides a 3D environment for MSC while maintaining MM cells in suspension culture (their natural 3D environment). This new approach leads to a much more physically realistic *in vitro* model.

Chemokine receptors and their ligands play a crucial role in the migration of plasma cells to and from the BM, as well as influencing their chemotaxis within the BM microenvironment [[31], [32], [33]]. Multiple myeloma cells express various chemokine receptors and secrete several chemokines, which contribute to cell homing, tumor growth, and progression [9,49]. The expression of CX3CR1 (also known as fractalkine) has recently been detected in human myeloma cell lines and is reported to induce osteoclast differentiation. In particular, the CX3CL1/CX3CR1 axis supports cooperation between multiple myeloma and osteoclast cells to promote adhesion of MM cells to bone extra cellular matrix (ECM), which is a required process for disease progression [29]. In our study, we have confirmed the expression of this chemokine and interestingly verified its over-expression as a consequence of co-culture with MSC. This suggests that the crosstalk between MSC and MM cells modulates the interaction with osteoclast cells and their differentiation, which in turn forms the basis of the MM-induced bone lesion owing to an imbalance between osteoblasts and osteoclasts [50,51].

Another remarkable effect on MM cells after co-culture is up-regulation of the interferon transcription factor 4 (IRF4). IRF4 is a member of the interferon regulatory family and has emerged as a master regulator in multiple myeloma, controlling a regulatory network to which MM cells are addicted, and that is essential for their survival. IRF4 directs B cell to plasma cell differentiation, immunoglobulin class switching and is overexpressed in cells derived from MM patients [30].

IRF4 up-regulation in MM cells after exposure to MSC is associated with greater secretion of IFN-γ in co-culture and suggests a pro-survival feedback. Moreover, the “inflammatory response” and “cellular proliferation” functional categories were identified as the most statistically significant ones associated with upregulated genes in co-cultured vs. mono-cultured MM cells.

Consistent with these results, upstream regulator analysis indicates that many cytokines are involved in the “inflammatory response” as potential upstream regulators. The “upstream analysis” tool enables a causal interpretation of the data [38] and confirmed that IL-6, previously reported to play an essential role in MM progression [52], and IL-10, an anti-inflammatory cytokine with important immunoregulatory functions [[53], [54], [55]], can be integrated within a mechanistic network that may account for the previously discussed gene up-regulation.

Furthermore, we have identified a synergistic mechanism between IL-6 and IL-10 that sustains the crosstalk-dependent proliferation of MM cells. Clinical studies confirm that both IL-6 and IL-10 are present at high concentrations in the serum of MM patients, and such serum concentrations strongly correlate with the disease stage [56,57]. The underlying mechanisms that account for these observations can be, at least in part, explained by our results.

Moreover, in accordance with the importance of Stat3 activation in IL-6 and IL-10 signaling [[43], [44], [45]], the array studies revealed higher levels of phospho-Stat3 in co-cultured cells. Stat3 is a multi-functional factor that is known to be important for regulating cell proliferation, differentiation, survival, and inflammatory response. Numerous studies suggest that aberrant activation of Stat3 promotes survival and proliferation of MM cells, and our results support these findings [[58], [59], [60]].

According to the literature, Multiple Myeloma (MM) patient-derived MSC differ in the production of cytokines compared to their normal counterparts [10,11]. It is unclear whether these abnormalities are acquired by MSC after exposure to MM cells or if they are primary. Previous results suggest that MM-MSC are intrinsically abnormal and remain as such despite being removed from the myeloma cell influence. However, an alternative explanation is that MM-MSC are only temporarily modified in response to MM cells [13,61,62]. Our results indicate that, after co-culture with MM cells, normal MSC can become *in vitro* MM-MSC, displaying some of the essential characteristics of patient-derived MM-MSC [63].

Although 3D models can mimic *in vivo* conditions better than 2D systems and provide valuable insights into the MM microenvironment, they do not fully resemble the physiological environment. For this reason, additional investigations testing the relevance of our findings on primary tumors are necessary, and should be the focus of future studies.

## 5 Conclusions

The present study offers significant new insights into the paracrine crosstalk between MSC and MM cells. Our findings suggest that IL-6/IL-10 signaling pathways may be crucial for the progression of MM, and therefore they may represent attractive therapeutic targets. We have shown that the inhibition of the synergistic IL-6/10 signaling pathway reduces the proliferation of MM cells.

Overall, our new 3D *in vitro* model based on a wholly synthetic, thermoresponsive hydrogel confirms that adjacent MSC and MM cells profoundly affect each other. This causes transcriptional activation and modifies the expression of specific signals, leading to greater proliferation of MM cells. Thus, we hypothesize that normal MSC revert to a “transformed” state *in vivo* which should be able to stimulate the disease progression. Further research is required to test this hypothesis and validate the efficacy of agents targeting MSC. This study has highlighted the potential scientific value in evaluating MM in the context of its BM microenvironment. In this perspective, future therapies that target not only tumor cells but also “accessory” cells could be very promising.

## Declaration of Competing Interest

The authors declare no competing financial interests or personal relationships that could have appeared to influence the work reported in this paper.
