# Supplementary material for: A worm gel-based 3D model to elucidate the paracrine interaction between multiple myeloma and mesenchymal stem cells
Source: Mater Today Bio. 2020 Jan 7;5:100040. doi: 10.1016/j.mtbio.2019.100040 (PMC7083757; doi:10.1016/j.mtbio.2019.100040)
Supplement: Multimedia component 1 [file mmc1.docx]

Supporting Information

**A worm gel-based 3D model to elucidate the paracrine interaction between multiple myeloma and mesenchymal stem cells**

*Renza Spelat ^a^, Federico Ferro ^a^, Paolo Contessotto ^a^, Nicholas J. Warren ^b^, Grazia Marsico ^a^, Steven P. Armes ^b^, and Abhay Pandit ^a*^*

^a^ CÚRAM, SFI Research Centre for Medical Devices,

National University of Ireland Galway,

Galway -H91 TK33,

Ireland

^b^ Department of Chemistry

University of Sheffield

Sheffield, South Yorkshire S3 7HF, United Kingdom

Corresponding author e-mail: abhay.pandit@nuigalway.ie

**
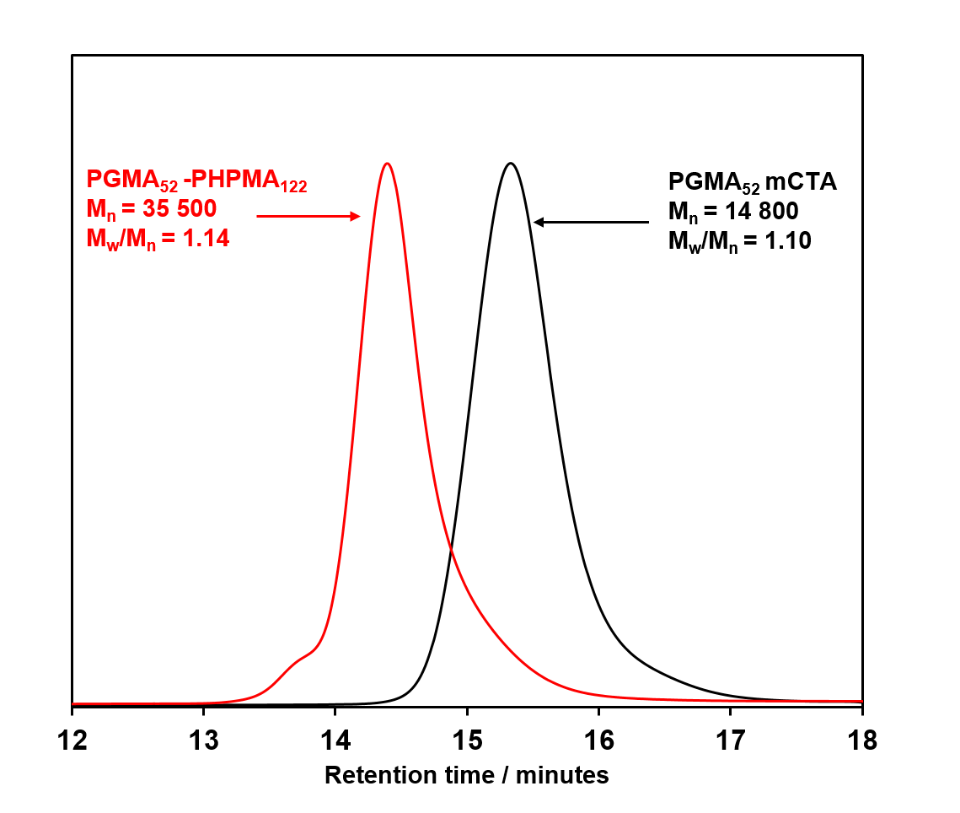
**

**Figure S1.** DMF GPC chromatograms obtained for the PGMA_52_ macro-CTA and the PGMA_52_-PHPMA_122_ diblock copolymers. *M*_n_ and *M*_w_/*M*_n_ were calculated by comparing to a series of near monodisperse PMMA standards.

**
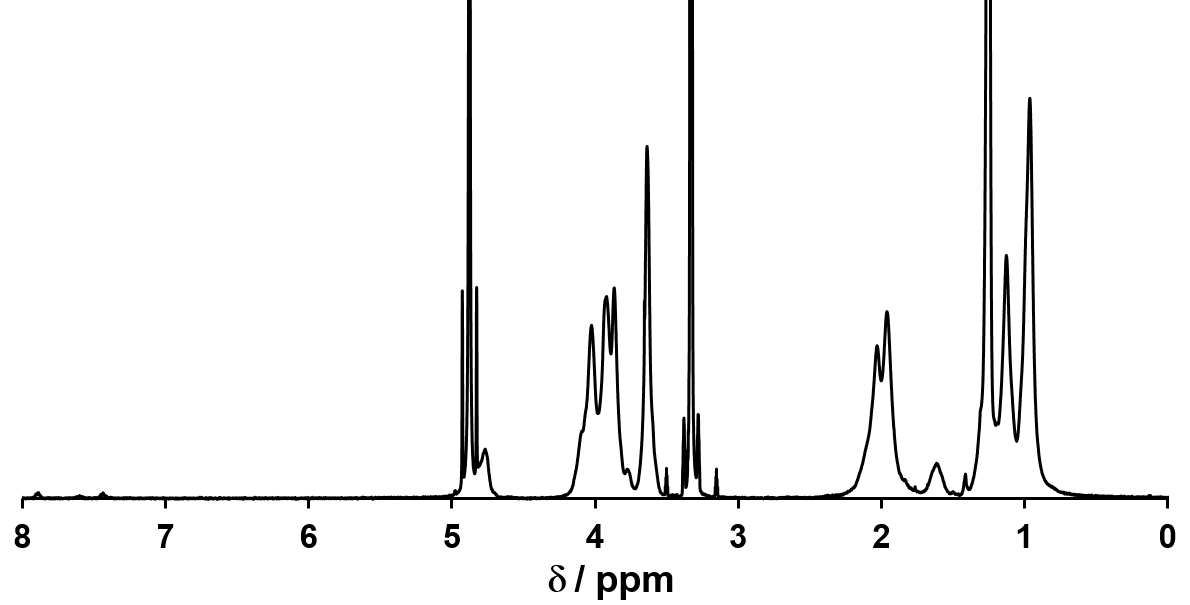
**

**Figure S2.** ^1^H NMR spectrum recorded for the PGMA_52_-PHPMA_122_ diblock copolymer after dissolving in CD_3_OD.

**
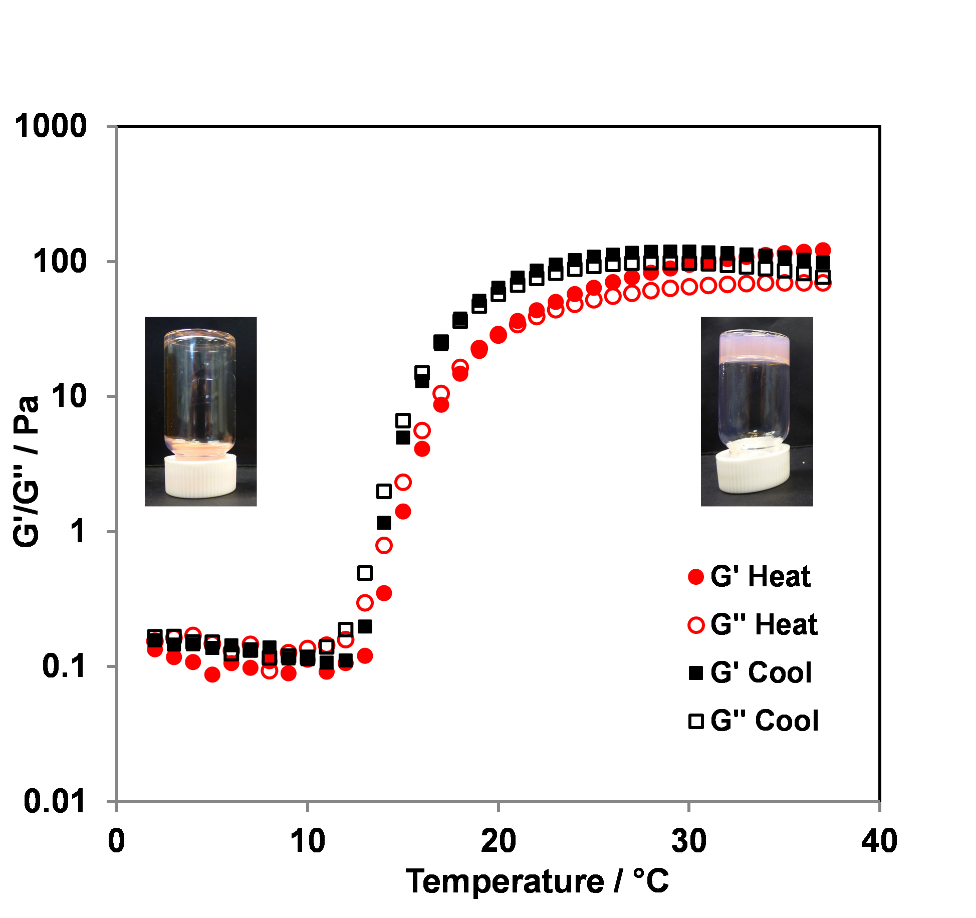
**

**Figure S3.** Temperature-dependent oscillatory rheology studies obtained on cooling (black data) and heating (red data) an aqueous dispersion of G_52_-H_122_ worms. Closed symbols represent G’ and open symbols represent G”. Oscillatory shear conditions: angular frequency = 1 rad s^-1^, applied strain amplitude = 1.0 %. Inset photographs of the same sample at 4°C (left) and 37°C (right).


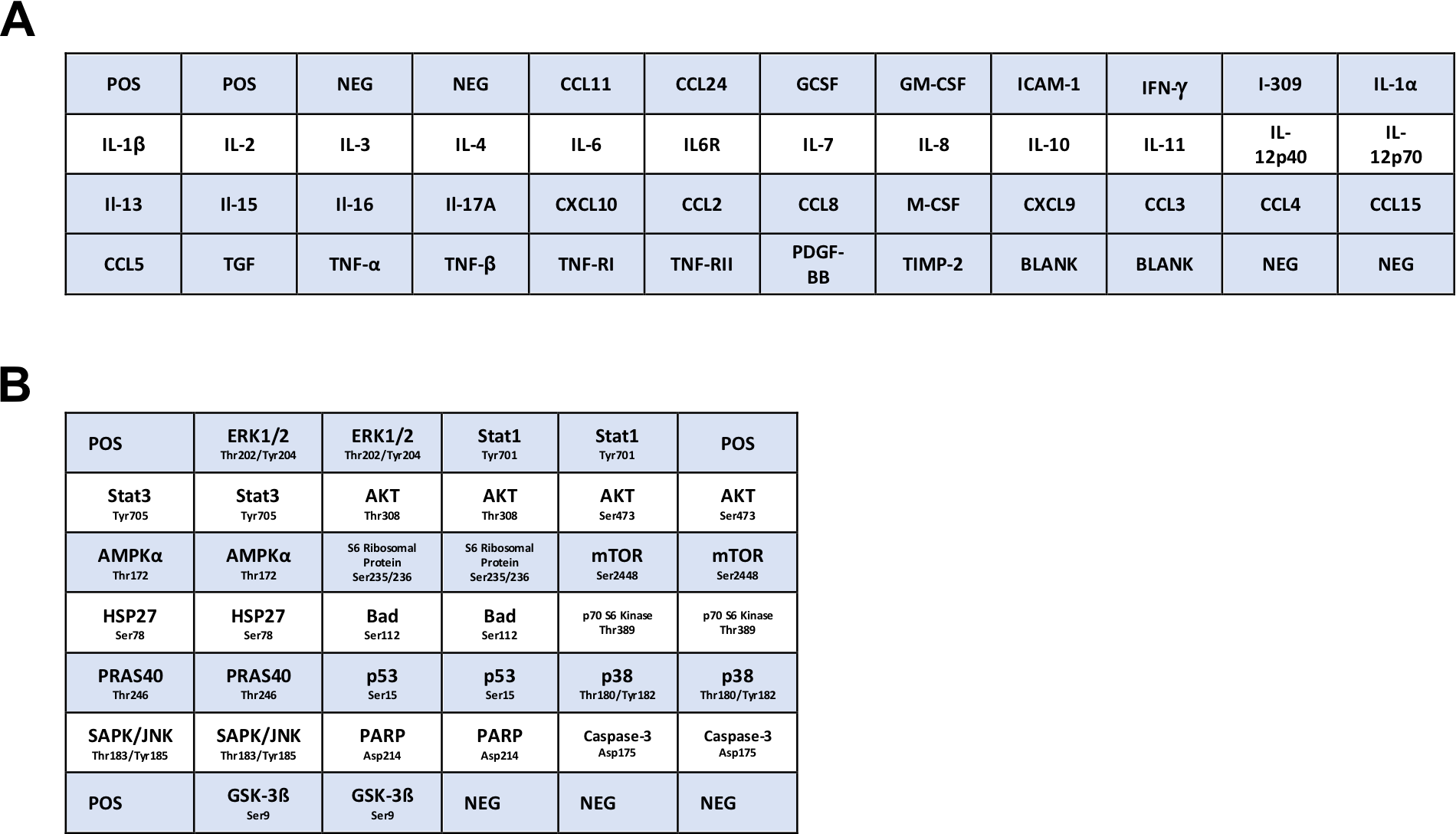


**Figure S4.** **A)** Cytokine protein array map. Each antibody is spotted in duplicate vertically. **B)** PathScan^®^ Intracellular Signaling array map. POS: positive control; NEG: negative control.

**Figure S5. MSC increase IL-6 and IL-10 production in co-culture. A)** IL-6 and **B)** IL-10 ELISA quantification in cell lysates from mono- or co-cultured MSC and RPMI-8226 cells after 48 hours of culture. 10^6^ cells were lysed in 100 µl of the lysis buffer provided with the kit after 48 hours of culture. **C)** IL-6 and **D)** IL-10 ELISA in mono- and co-cultured MM1S cells. (N = 3 independent experiments, **p* < 0.05, * * *p* < 0.01, * * * *p* < 0.001).


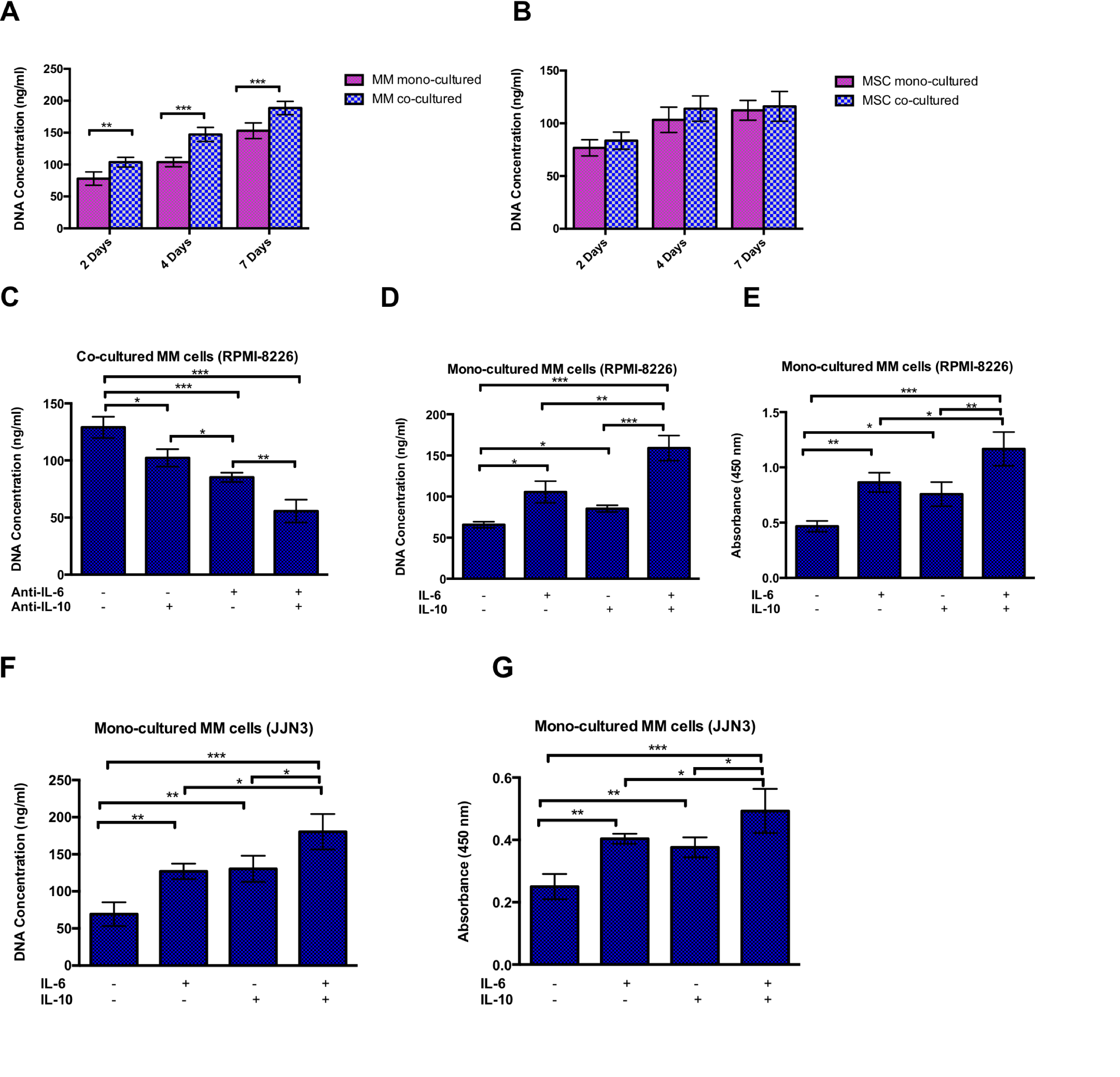


**Figure S6. Influence of co-culture, IL-6 and IL-10 treatments on MM cells proliferation. A)** Comparison of proliferation determined by PicoGreen^®^ assay in mono- and co-cultured MM and **B)** MSC cells. **C)** PicoGreen^®^ assay of co-cultured MM cells (RPMI-8226) treated with IL-6 and IL-10 blocking antibodies and of **D)** mono-cultured MM cells (RPMI-8226) treated with IL-6 (100 ng/ml), IL-10 (100 ng/ml) or a combination of them determined by PicoGreen^®^ and **E)** BrdU (Bromodeoxyuridine) assays **F)** PicoGreen^®^ assay and **G)** BrdU assay on JJN3 MM cells treated as RPMI-8226 with IL-6 and IL-10. (N = 3 independent experiments, **p* < 0.05, * * *p* < 0.01, * * * *p* < 0.001).
